# Identifying shared genetic loci between coronavirus disease 2019 and cardiovascular diseases based on cross-trait meta-analysis

**DOI:** 10.3389/fmicb.2022.993933

**Published:** 2022-09-15

**Authors:** Hongping Guo, Tong Li, Haiyang Wen

**Affiliations:** ^1^School of Mathematics and Statistics, Hubei Normal University, Huangshi, China; ^2^School of Computational Science and Electronics, Hunan Institute of Engineering, Xiangtan, China

**Keywords:** COVID-19, cardiovascular diseases, shared genetics, meta-analysis, GWAS

## Abstract

People with coronavirus disease 2019 (COVID-19) have different mortality or severity, and this clinical outcome is thought to be mainly attributed to comorbid cardiovascular diseases. However, genetic loci jointly influencing COVID-19 and cardiovascular disorders remain largely unknown. To identify shared genetic loci between COVID-19 and cardiac traits, we conducted a genome-wide cross-trait meta-analysis. Firstly, from eight cardiovascular disorders, we found positive genetic correlations between COVID-19 and coronary artery disease (CAD, *R*_*g*_ = 0.4075, *P* = 0.0031), type 2 diabetes (T2D, *R*_*g*_ = 0.2320, *P* = 0.0043), obesity (OBE, *R*_*g*_ = 0.3451, *P* = 0.0061), as well as hypertension (HTN, *R*_*g*_ = 0.233, *P* = 0.0026). Secondly, we detected 10 shared genetic loci between COVID-19 and CAD, 3 loci between COVID-19 and T2D, 5 loci between COVID-19 and OBE, and 21 loci between COVID-19 and HTN, respectively. These shared genetic loci were enriched in signaling pathways and secretion pathways. In addition, Mendelian randomization analysis revealed significant causal effect of COVID-19 on CAD, OBE and HTN. Our results have revealed the genetic architecture shared by COVID-19 and CVD, and will help to shed light on the molecular mechanisms underlying the associations between COVID-19 and cardiac traits.

## Introduction

The coronavirus disease 2019 (COVID-19) arises from severe acute respiratory syndrome coronavirus 2 (SARS-CoV-2) infection, and it rapidly outbreak since November 2019 and recently become a public health emergency of international concern (The Severe Covid-19 Gwas Group, 2020). Up to now, there have been more than 170 million confirmed cases and nearly 3.9 million deaths globally. However, its etiology is not fully understood.

People with COVID-19 have different mortality or severity, and the clinical outcome are worse in patients with cardiovascular related disorders, which suggests the comorbidity of COVID-19 and cardiovascular diseases (CVD) (Guan et al., 2020b). More evidences showed the concordant result (Guan et al., 2020a; Ruan et al., 2020; Sisnieguez et al., 2020; Wang et al., 2020; Yang et al., 2020). On the one hand, it was reported that hypertension (21.1%) and diabetes (9.7%) ranked as the top two most prevalent comorbidities for COVID-19 (Wang et al., 2020). The odd ratios of hypertension (2.36) and coronary heart disease (3.42) were larger than 1 when comparing severe COVID-19 patients to non-severe cases (Yang et al., 2020). On the one hand, genome-wide association studies (GWAS) have identified several associated-variants involved in COVID-19 and cardiovascular disease-related traits. For example, a gene known as *ERI3* has been associated with COVID-19 related mortality, coronary artery disease and type 2 diabetes (MacArthur et al., 2017). Moreover, COVID-19 cardiovascular epidemiology showed that nearly 12% of COVID-19 cases have been found to have sustained cardiac injuries, COVID-19 might have a direct and indirect effect on the cardiovascular system (Tajbakhsh et al., 2021). The etiologic agent of COVID-19 can infect the heart, vascular tissues, and circulating cells through the host cell receptor for the viral spike protein (Chung et al., 2021). All the above studies lead us to wonder whether the comorbidity between COVID-19 and CVD is due to the potential shared genetic factors. However, there is few genetic study to reveal the common genetic architecture between COVID-19 and CVD. To this end, the goal of this study was to identify genetic loci shared between COVID-19 and cardiac traits by conducting a large-scale genome-wide cross-trait meta-analysis, and provide more knowledge about common molecular mechanisms of them.

Our study mainly includes three parts. Firstly, we estimated both the overall and local genetic correlation between COVID-19 and eight cardiac traits, including coronary artery disease (CAD), type 2 diabetes (T2D), hypertension (HTN), obesity (OBE), high-density lipoproteins (HDL), low-density lipoproteins (LDL), triglycerides (TC), and total cholesterol (TG). Secondly, we carried out a large-scale cross-trait meta-analysis to identify shared genetic loci between trait pairs that showed significant genetic correlation in the first part of the study. Finally, we conducted transcriptome-wide association study (TWAS), pathway enrichment analysis and Mendelian randomization (MR) analysis to obtain more biological insight.

**FIGURE 1:**
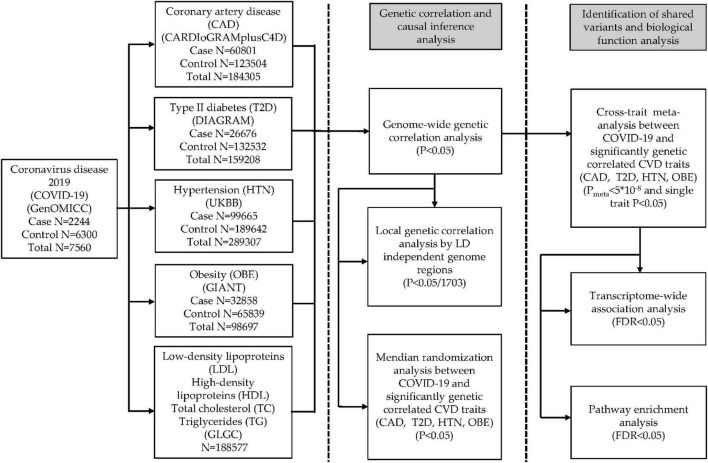
Flow chart of the present work.

The overall study design is shown in Figure 1.

## Materials and methods

### Data sources

The GWAS summary statistic for COVID-19 was extracted from the Genetics of Mortality in Critical Care (GenOMICC) study, which performed GWAS on 2244 critically ill patients with COVID-19 in 208 UK intensive care units (Pairo-Castineira et al., 2021). We downloaded the summary statistic with European cases vs UK Biobank controls in this study. We also retrieved the summary statistics of eight cardiac traits in the following public available datasets. The summary statistic for CAD was from the Coronary ARtery DIsease Genome Wide Replication and Meta-analysis plus The Coronary Artery Disease Genetics (CARDIoGRAMplusC4D) Consortium (60,801 cases and 123,504 controls) (Nikpay et al., 2015). The summary statistic for T2D was from the Diabetes Genetics Replication and Meta-Analysis (DIAGRAM) Consortium (26,676 cases and 132,532 controls) (Scott et al., 2017). The summary statistic for OBE was from the Genetic Investigation of ANthropometric Traits (GIANT) Consortium (32,858 cases and 65,839 controls) (Berndt et al., 2013). The summary statistic for HTN was from the Genome wide association study ATLAS (GWASATLAS) database (99,665 cases and 189,642 controls) (Watanabe et al., 2019). The summary statistics for four lipid traits (LDL, HDL, TC, and TG) were from the Global Lipids Genetics Consortium (GLGC) Consortium (188,577 samples) (Willer et al., 2013). The details of each summary statistic dataset are provided in Supplementary Table 1.

### Genome-wide genetic correlation analysis

We employed the high-definition likelihood methodology (Ning et al., 2020) to estimate the genetic correlation between COVID-19 and eight cardiac traits. This approach provides more accurate estimation by fully accounting for linkage disequilibrium (LD) information across the whole genome. The χ^2^ statistic of single nucleotide polymorphisms (SNPs) in high LD regions is higher than that of those in low LD regions, and similar results are observed by replacing one study test statistic with the product of two z-scores in the study. We used the reference panel with imputed HapMap3 SNPs, which are based on genotypes in UK Biobank.

### Local genetic correlation

We applied ρ-HESS (Shi et al., 2017) to investigate whether COVID-19 and cardiac traits show local genetic correlation. ρ-HESS quantifies the correlation between traits at each LD-independent region of the genome due to genetic variation. A total of approximately 1.5 Mb was used for estimating local genetic heritabilities and genetic covariances from independent LD blocks. We chose the cardiac traits that showed significant genetic correlation with COVID-19 in this analysis, thus, four pairs of traits were included (COVID-19 and CAD, COVID-19 and T2D, COVID-19 and OBE, COVID-19 and HTN). Notice that we removed the empty loci (with no SNP in it) in each local region in ρ-HESS.

### Cross-trait meta-analysis

We conducted a large-scale cross-trait meta-analysis to identify genetic loci shared between severe COVID-19 and cardiac traits that showed significant genetic correlation, using PLEIO framework (Lee et al., 2021). PLEIO is a summary-statistics approach to mapping pleiotropic loci in a multiple trait analysis, either binary, quantitative, independent or correlated traits. Besides, this method can maximize power by adequately modeling the genetic architectures (genetic correlation and heritability) and control false positive rate by accounting for environmental correlation. SNPs with *P*_meta_ < 5 × 10^–8^ and trait-specific *P* < 0.05 were considered to be significant for both traits. We performed the operations on a computer of Intel Xeon E5-2695 CPU 2.10 GHz. For each disease pair, it will waste 8–10 mins for the standardization of raw summary statistics first, and then about 2 mins for the identification of pleiotropic loci with PLEIO.

The independent loci were identified using the clumping function of PLINK (version 1.9) tool (Purcell et al., 2007) with clumping parameters *p*_1_ = 5 × 10^–8^, *p*_2_ = 1 × 10^–5^, *r*^2^ = 0.1, and kb = 500, that is, SNPs with *p* value less than 1 × 10^–5^, *r*^2^ greater than 0.1 and distance less than 500 kb from the peak will be assigned to the clump with that peak. Distance to the nearest gene was calculated using NCBI human genome build37 gene annotation.

### Transcriptome-wide association study

We performed transcriptome-wide association study (TWAS) to detect gene expression associations in specific tissues for COVID-19 and cardiac traits, using FUSION software (Gusev et al., 2016) based on 43 Genotype-Tissue Expression Project (GTEx: version 6) tissue expression weights. FUSION is a powerful strategy that uses cis-regulated gene expression measurements to identify genes associated with complex traits through large-scale summary statistics. TWAS *p* values for each trait were corrected for multiple testing by using Benjamini-Hochberg’s False Discovery Rate (FDR) procedure (FDR < 0.05).

### Pathway enrichment analysis

To obtain biological insight for shared risk genes that were identified from cross-trait meta-analysis, we used Enrichr tool (Kuleshov et al., 2016) to perform Kyoto Encyclopedia of Genes and Genomes (KEGG) pathway enrichment analysis. The Benjamini-Hochberg procedure was used on *p* value to account for multiple testing.

### Mendelian randomization analysis

In order to examine the causal relationships between COVID-19 and cardiac traits, we conducted MR analysis using MR-PRESSO test (Verbanck et al., 2018). The MR-PRESSO method estimates exposure effects in multi-instrument MR using SNPs significantly associated with exposure, as well as horizontal pleiotropy in multi-instrument MR utilizing summary statistics. Instruments were constructed using LD-independent SNPs with *p* values lower than 5 × 10^–8^.

## Results

### Overall and local genetic correlations between coronavirus disease 2019 and cardiac traits

We estimated the genetic correlation between COVID-19 and eight cardiac traits using high-definition likelihood method. Four out of eight cardiac traits showed strong and significant genetic correlation with COVID-19. There was the strongest genetic correlation between COVID-19 and CAD (*R*_*g*_ = 0.4075, *P* = 0.0031), followed by T2D and HTN in a similar magnitude (*R*_*g*_ = 0.232, *P* = 0.0043 and *R*_*g*_ = 0.233, *P* = 0.0026, respectively). Moreover, a positive genetic correlation was also found with COVID-19 in OBE (*R*_*g*_ = 0.3451, *P* = 0.0061). However, no significant genetic correlation was found between COVID-19 and four lipid traits (LDL, HDL, TC, and TG). The detailed results of genetic correlation are displayed in Table 1.

**TABLE 1:** Genetic correlation between coronavirus disease 2019 and cardiac traits.

Phenotype 1	Phenotype 2	*R*_g_	SE	*P*
COVID-19	CAD	0.4075	0.1379	0.0031
	T2D	0.232	0.1147	0.0043
	OBE	0.3451	0.1259	0.0061
	HTN	0.233	0.0774	0.0026
	LDL	0.0335	0.1058	0.7510
	HDL	−0.1923	0.1169	0.1000
	TC	0.0292	0.0852	0.7320
	TG	0.1928	0.1049	0.0661

R_g_, genetic correlation estimate; SE, standard error of genetic correlation; COVID-19, coronavirus disease 2019; CAD, coronary artery disease; T2D, type 2 diabetes; OBE, obesity; HTN, hypertension; LDL, low-density lipoproteins; HDL, high-density lipoproteins; TC, total cholesterol; TG, triglycerides.

**FIGURE 2:**
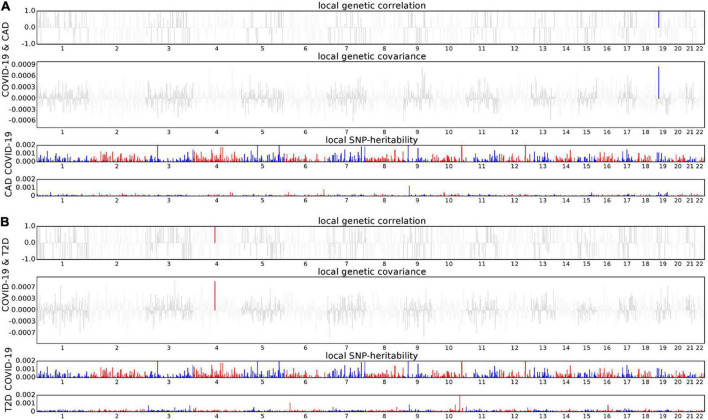
Local genetic correlation and local SNP-heritability between COVID-19 and CAD **(A)**, T2D **(B)**, respectively. For each subfigure, the top part represents local genetic correlation, the middle part represents local genetic covariance, and blue or red highlights indicate significant local genetic correlation and covariance after multiple testing correction, the bottom part represents local SNP heritability for each trait.

Due to the significant genetic correlation between COVID-19 and four cardiac traits (CAD, T2D, OBE, and HTN), we conducted ρ-HESS to explore whether there is a genetic correlation between COVID-19 and cardiac traits in certain regions of the genome. Result of the COVID-19/CAD trait pair showed that the 19p13.2 region (chromosome 19: 9238393-11284028) had strong local genetic correlation (*P* = 3.76 × 10^–6^). Besides, result of the COVID-19/T2D trait pair showed strong local genetic correlation (*P* = 1.39 × 10^–7^) in the 4q21.23 region (chromosome 4: 83372593-84799656). We did not find significant local genetic correlations for neither COVID-19/HTN nor COVID-19/OBE trait pair (Figure 2).

### Cross-trait meta-analysis results between coronavirus disease 2019 and cardiac traits

We performed a large-scale genome-wide cross-trait meta-analysis to improve the statistical power to identify shared genetic loci between COVID-19 and four cardiac traits that show significant genetic correlations. We considered SNPs with *P*_meta_ < 5 × 10^–8^ and trait-specific *P* < 0.05 to be significant for both COVID-19 and cardiac traits. Based on these criteria, we identified 39 independent loci significantly associated with COVID-19 and cardiac traits, of which eight loci failed to be detected in trait-specific GWAS of COVID-19 and cardiac traits (Tables 2, 3).

**TABLE 2:** Cross-trait meta-analysis results between coronavirus disease 2019 and CAD, T2D, and OBE (*P*_meta_ < 5 × 10^–8^; single trait *P* < 0.05).

Traits	SNP	Genome position	Eff. alle.	Ref. alle.	MAF	COVID-19 *P*	Cardiac trait *P*	Meta OR	Meta *P*	Genes within clumping region
CAD	rs1122608	chr19:10891239–11177408	T	G	0.259	0.017	2.73×10^−11^	1.08	2.23×10^−13^	*C19orf38, C19orf52, CARM1, DNM2, SMARCA4, TMED1*, and *YIPF2*
	rs495828	chr9:136154867–136154867	T	G	0.217	0.019	1.29×10^−10^	0.93	1.19×10^−12^	ABO*
	rs6705971	chr2:85755357–85809989	C	A	0.468	0.004	4.52×10^−10^	0.94	3.23×10^−12^	*GGCX, MAT2A*, and *VAMP8*
	rs6694817	chr1:154401972–154426264	T	C	0.425	0.037	2.96×10^−9^	0.95	1.59×10^−10^	*IL6R*
	rs17678683	chr2:145286559–145286559	G	T	0.091	0.035	3.00×10^−9^	0.91	2.61×10^−10^	LINC01412*
	rs2437935	chr10:44752268–44793299	G	A	0.358	0.014	6.98×10^−9^	1.06	3.46×10^−10^	*C10orf142*
	rs4691707	chr4:156441314–156441314	G	A	0.348	0.003	5.95×10^−7^	0.95	6.15×10^−9^	MTND1P22*
	rs17612742	chr4:148401190–148414651	C	T	0.138	0.039	1.61×10^−7^	0.93	1.29×10^−8^	*EDNRA*
	rs3002124	chr1:222748085–222748085	G	A	0.293	0.011	7.98×10^−7^	1.06	2.84×10^−8^	*TAF1A*
	rs17251589	chr19:41756085–41756906	C	T	0.119	0.025	3.29×10^−7^	0.92	3.44×10^−8^	*AXL*
T2D	rs6446490	chr4:6323465–6325086	G	A	0.451	1.00×10^−4^	1.70×10^−10^	1.08	2.30×10^−13^	*PPP2R2C*
	rs6798189	chr3:123095312–123095312	G	A	0.266	0.040	1.30×10^−10^	0.91	1.08×10^−10^	*ADCY5*
	rs1359790	chr13:80717156–80717156	G	A	0.288	0.011	1.40×10^−8^	0.92	3.89×10^−9^	Intergenic region
OBE	rs16917237	chr11:27702383–27702383	T	G	0.204	0.048	3.60×10^−11^	1.11	8.07×10^−14^	*BDNF*
	rs3136673	chr3:46031957–46272440	T	C	0.086	6.87×10^−9^	0.0093	1.06	5.90×10^−10^	*CCR1, FYCO1*, and *XCR1*
	rs7189927	chr16:28913787–28922149	C	T	0.356	0.013	3.40×10^−7^	1.07	6.07×10^−10^	*ATP2A1* and *RABEP2*
	rs1541984	chr2:25079770–25100328	G	A	0.428	0.049	1.80×10^−8^	1.07	7.42×10^−10^	*ADCY3*
	rs1766530	chr6:97576742–97576742	A	G	0.314	2.40×10^−3^	6.90×10^−6^	1.06	6.42×10^−9^	*KLHL32* and *MIR548H3*

*The nearest genes to these loci. COVID-19, coronavirus disease 2019; CAD, coronary artery disease; T2D, type 2 diabetes; OBE, obesity; SNP, single nucleotide polymorphisms; chr, chromosome; Eff. alle., effect allele; Ref. alle., reference allele; MAF, minor allele frequency; OR, odds ratios.

**TABLE 3:** Cross-trait meta-analysis result between coronavirus disease 2019 and HTN (*P*_meta_ < 5 × 10^–8^; single trait *P* < 0.05).

Traits	SNP	Genome position	Eff. alle.	Ref. alle.	MAF	COVID-19 *P*	HTN *P*	Meta OR	Meta *P*	Genes within clumping region
HTN	rs1401982	chr12:89989599–90441215	G	A	0.413	0.0056	8.50×10^−28^	0.94	4.32×10^−32^	*ATP2B1*
	rs35441	chr12:115552499–115553115	T	C	0.383	0.0256	3.04×10^−25^	0.94	1.19×10^−28^	Intergenic region
	rs2137320	chr11:1884342–1884342	A	G	0.387	0.022	3.82×10^−23^	1.06	1.34×10^−25^	*LSP1*
	rs17080093	chr6:150989698–151027008	T	C	0.069	0.0232	3.86×10^−20^	0.90	3.23×10^−22^	*PLEKHG1*
	rs936228	chr15:75131661–75225415	T	C	0.277	0.0081	9.97×10^−19^	1.05	6.33×10^−21^	*COX5A, FAM219B, MPI, SCAMP2*, and *ULK3*
	rs3942852	chr11:48028343–48136990	C	T	0.209	0.0329	9.92×10^−19^	0.94	7.33×10^−20^	*PTPRJ*
	rs6055976	chr20:8629857–8630692	A	C	0.229	0.0442	1.56×10^−17^	0.94	1.25×10^−18^	*PLCB1*
	rs2279500	chr1:113230394–113248791	T	C	0.167	0.0017	1.31×10^−12^	0.95	1.87×10^−13^	*MOV10* and *RHOC*
	rs17419291	chr5:87780432–88178683	C	T	0.086	0.0079	1.29×10^−12^	0.93	3.20×10^−13^	*MEF2C*
	rs2242261	chr11:47038220–47282024	G	T	0.155	0.0424	4.34×10^−13^	0.94	1.05×10^−12^	*ACP2, ARFGAP2, C11orf49, DDB2, NR1H3*, and *PACSIN3*
	rs495828	chr9:136139265–136154867	T	G	0.217	0.0187	1.11×10^−12^	0.95	1.61×10^−12^	*ABO*
	rs7716011	chr5:157525853–157525853	G	T	0.252	0.0366	6.21×10^−12^	1.04	2.48×10^−11^	LINC02056*
	rs3744251	chr17:7760983–7760983	A	G	0.076	0.0483	3.94×10^−11^	1.07	1.84×10^−10^	*NAA38*
	rs1918966	chr3:169098791–169181582	A	G	0.455	0.0342	5.56×10^−11^	1.04	2.04×10^−10^	*MECOM*
	rs4691707	chr4:156441314–156499985	G	A	0.348	0.0025	1.36×10^−9^	1.04	3.03×10^−10^	MTND1P22*
	rs11858678	chr15:41353079–41542591	G	A	0.428	0.0362	1.00×10^−10^	1.04	3.64×10^−10^	*CHP1, EXD1*, and *INO80*
	rs7254154	chr19:17169936–17178119	C	A	0.410	0.0258	7.99×10^−9^	1.03	1.54×10^−8^	*HAUS8*
	rs2228615	chr19:10403368–10403368	A	G	0.377	4.45×10^−6^	7.67×10^−6^	0.97	3.41×10^−8^	*ICAM5*
	rs11707155	chr3:53608306–53608306	G	A	0.038	0.0183	3.14×10^−8^	1.09	4.23×10^−8^	*CACNA1D*
	rs3809278	chr12:111725185–111725185	A	C	0.130	0.0222	2.78×10^−8^	0.95	4.31×10^−8^	*CUX2*
	rs2348427	chr4:111414399–111414399	T	C	0.447	0.0016	5.00×10^−7^	1.03	4.98×10^−8^	*ENPEP*

*The nearest genes to these loci. COVID-19, coronavirus disease 2019; HTN, hypertension; SNP, single nucleotide polymorphisms; chr, chromosome; Eff. alle., effect allele; Ref. alle., reference allele; MAF, minor allele frequency; OR, odds ratios.

We observed two overlapped significant loci in the cross-trait meta-analysis of COVID-19/CAD and COVID-19/HTN. The first association signal was 9q34.2 (index SNP: rs495828, *P*_meta_ = 1.19 × 10^–12^ for COVID-19/CAD; *P*_meta_ = 1.61 × 10^–12^ for COVID-19/HTN). This locus was located at the ABO blood group, which contributed to the immunopathogenesis of SARS-CoV-infection (The Severe Covid-19 Gwas Group, 2020). Similarly, it was concluded that group A individuals had a higher risk of COVID-19 respiratory failure while group O individuals had a protective effect *via* blood type-specific analysis (Deleers et al., 2021). The other locus (index SNP: rs4691707, *P*_meta_ = 6.15 × 10^–9^ for COVID-19/CAD; *P*_meta_ = 3.03 × 10^–10^ for COVID-19/HTN) was in the intergenic region closet to the *MTND1P22* gene, which may have a role in transcription regulation.

In addition to rs495828 and rs4691707, a further eight loci were identified to be associated with COVID-19 and CAD (Table 2). The strongest association signal (index SNP: rs1122608, *P*_meta_ = 2.23 × 10^–13^) was found near gene *SMARCA4* on chromosome 19, which was previously reported to regulate atherosclerosis (Ma et al., 2019) and play a protective role to against the risk of HTN (Xiong et al., 2014).

Three loci were identified in a cross-trait meta-analysis of COVID-19 and T2D (Table 2). The first locus (index SNP: rs6446490, *P*_meta_ = 2.30 × 10^–13^) was mapped on *PPP2R2C*, a gene that increased insulin resistance (Daily et al., 2019). The second locus represented by rs6798189 (*P*_meta_ = 1.08 × 10^–10^) was mapped on *ADCY5*, a gene coupled glucose to insulin secretion in human islets (Hodson et al., 2014). The third locus (index SNP: rs1359790, *P*_meta_ = 3.89 × 10^–9^) located in intergenic region, which was previously reported to be associated with T2D (Flannick et al., 2019).

We also found five significant loci that were associated with both COVID-19 and OBE (Table 2). The top locus (index SNP: rs16917237, *P*_meta_ = 8.07 × 10^–14^) was mapped on *BDNF*, a gene was not only associated with body mass index but also CAD (Winkler et al., 2015; van der Harst and Verweij, 2018). The second locus (index SNP: rs3136673, *P*_meta_ = 5.90 × 10^–10^) was originally significant associated with COVID-19 (*P* = 6.87 × 10^–9^), the mapped gene *CCR1* involved in heart and blood communication in cardiac diseases.

In the cross-trait meta-analysis of COVID-19 and HTN, we identified 21 significant loci (Table 3). One of the most important loci is characterized by the *ATP2B1* gene (index SNP: rs1401982, *P*_meta_ = 4.32 × 10^–32^), which plays a key role in regulating blood pressure by altering calcium handling and vasoconstriction in vascular smooth muscle cells (Wain et al., 2011).

### Results of transcriptome-wide association analysis, pathway enrichment analysis, and Mendelian randomization analysis

To identify association between COVID-19 and cardiac traits with gene expression in specific tissue, we performed TWAS in 43 GTEx tissues. A total of 20 gene-tissue pairs were significantly associated with COVID-19, in addition to 263 gene-tissue pairs with CAD, 142 gene-tissue pairs with T2D, 2030 gene-tissue pairs with HTN, and 256 gene-tissue pairs with OBE (Supplementary Tables 2–6). There is no gene-tissue pair overlapped between COVID-19 and the four cardiac traits in TWAS.

**FIGURE 3:**
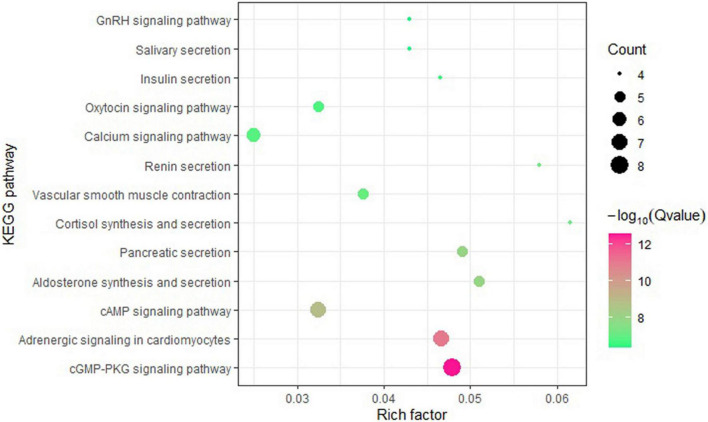
Bubble chart of enrichment analysis of shared genes.

To investigate the biological pathways represented by shared genes, we assessed enrichment of shared genes between COVID-19 and cardiac traits. KEGG pathway enrichment analysis revealed cGMP-PKG signaling pathway as the most significant pathway, as well as other signaling pathways and secretion pathways (Figure 3).

We identified three significant causal relationships by using MR-PRESSO test, including the effect of COVID-19 on CAD (causal estimate = 0.0045, *P* = 3.70 × 10^–6^), OBE (causal estimate = 0.0494, *P* = 1.86 × 10^–4^), and HTN (causal estimate = 0.0019, *P* = 1.20 × 10^–6^). However, we did not observed causal effect of COVID-19 on T2D (causal estimate = –0.0026, *P* = 0.2281; Supplementary Table 7).

## Discussion

To the best of our knowledge, this is the study to identify shared genetic architecture between COVID-19 and cardiac traits. Specifically, we found substantial and significant genetic correlation between COVID-19 and CAD, T2D, OBE, and HTN. These findings are consistent with the study which estimated the genetic correlation by LD score regression method (Chang et al., 2021), and further confirmed the fact that patients with certain underlying medical conditions (such as CAD, T2D, OBE, and HTN) are at increased risk for poor outcome in COVID-19 (Richardson et al., 2020).

In the original GWAS summary statistics, there were hundreds to thousands of significant loci (*P* < 5 × 10^–8^) in each of these diseases. However, no shared genetic locus was found between COVID-19 and any of the four cardiac traits. After cross-trait meta-analysis, we identified 10 shared loci between COVID-19 and CAD, three shared loci between COVID-19 and T2D, five shared loci between COVID-19 and OBE, and 21 shared locus between COVID-19 and HTN. This series of comparative data highlights the superiority of cross-trait meta-analysis. These shared genetic loci could be used to predict the occurrence of COVID-19 as well as the abnormal cardiac traits. In addition, we identified eight loci that failed to reach significance in trait-specific GWAS, demonstrating cross-trait meta-analysis’ excellent statistical power similarly.

We performed GWAS-Catalog analysis to understand whether the shared genes have been reported in the previous studies (Supplementary Table 8). Gene ABO, mapped by the locus rs495828 in 9q34.2 region, was reported to be associated with COVID-19, CAD, OBE and HTN (Covid-19 Host Genetics Initiative., 2021). Additionally three genes (CCR1, FYCO1, and XCR1) were not only associated with COVID-19, but also at least two cardiac traits (Shelton et al., 2021). Beyond them, other shared genes were newly found. In terms of gene function, ABO, which determines blood type, may affect COVID-19 disease severity, but there was no evidence to confirm ABO blood group influences risk of COVID-19 infection or outcome (Lehrer and Rheinstein, 2021). Genes CCR1, FYCO1, and XCR1 were involved in T-cell and dendritic-cell function (Kaser, 2020).

In the local genetic correlation analysis between COVID-19 and cardiac traits that showed significant genetic correlation, we found that *SMARCA4* region to have genetic correlation between COVID-19 and CAD, which was also identified by cross-trait meta-analysis. *SMARCA4* is a well-known gene associated with CAD, and it mediated nucleosome remodeling which was considered another epigenetic mechanism that can affect the course of COVID-19 (Peng et al., 2020; Shirvaliloo, 2021). Moreover, we also identified *HELQ* region to be significantly associated with COVID-19 and T2D. *HELQ* is predominantly known for its ATP-dependent helicase activity and participation in DNA repair.

Post-GWAS function analyses provided biological insights into the shared genes between COVID-19 and four cardiac traits. In TWAS analysis, we detected 20 significant gene-tissue pair associated with COVID-19, 263 with CAD, 142 with T2D, 256 with OBE and 2030 with HTN. Of these, none of the gene-tissue pair significantly associated with COVID-19 and cardiac traits. In addition, we also performed GTEx tissue enrichment analysis, and did not identify any enrichment signal in tissues. These results suggest that the distribution of pleiotropic genes between COVID-19 and cardiac traits is scattered and not limited to a specific tissue. Moreover, KEGG pathway enrichment analysis showed that the shared genes enriched in some signaling pathways and secretion pathways, such as cGMP-PKG signaling pathway, pancreatic secretion and insulin secretion. The recent studies reported that signaling pathways significantly related to COVID-19 (Messina et al., 2021; Wang et al., 2021), and secretion pathways significantly related to cardiovascular diseases (Chae and Kwon, 2019).

Our MR analysis showed causal effect of COVID-19 on CAD, OBE, and HTN, these findings supported the idea that the genetic correlation of polygenic diseases may be due to both causality and pleiotropy (van Rheenen et al., 2019). Moreover, there is no causal relationship between COVID-19 and T2D, this result indicated the shared genetic effect between COVID-19 and T2D is more likely to be pleiotropic effect, rather than causal effect or mechanism.

As well as genetic factors, environmental factors and lifestyle also play an important role in the comorbidity of COVID-19 and cardiac traits. Although there are many studies on screening anti-SARS-CoV-2 drugs and discoverying potential therapeutic drugs for COVID-19 (Zhou et al., 2020; Peng et al., 2021; Shen et al., 2022; Tian et al., 2022), home quarantine and staying away from infection prevention, vaccination, appropriate immunomodulatory diet and drugs that modulate cardiovascular system are currently the most effective approach to prevention (Lotfi et al., 2020).

We also acknowledge some limitations of our work. First, we restricted the analysis to the participants of European ancestors to avoid population stratification, so the findings may not be applicable to general populations. Second, we observed some positive genetic correlation between COVID-19 and TG as well as negative genetic correlation between COVID-19 and HDL, but they failed to reach the standard significant level. The genetic relationship between severe COVID-19 and lipid traits deserves further study. Third, although large sample cohorts were used in this study, we did not perform replication with other COVID-19 cohorts, which would be meaningful to confirm our findings.

## Conclusion

In conclusion, our genome-wide cross-trait meta-analysis confirmed the association between COVID-19 and cardiovascular disorders. Investigation of the shared genetic loci between COVID-19 and cardiac traits can be helpful to understand the common biological mechanisms underlying the comorbidity.

## Data availability statement

The original contributions presented in this study are included in the article/Supplementary material, further inquiries can be directed to the corresponding author.

## Author contributions

HG conceived the project, conducted data analysis, and wrote the manuscript. HW performed the methodology and software. TL participated in the discussion and revised the manuscript. All authors contributed to the article and approved the submitted version.
